# Impact of the Curing Temperature on the Manufacturing Process of Multi-Nanoparticle-Reinforced Epoxy Matrix Composites

**DOI:** 10.3390/ma17081930

**Published:** 2024-04-22

**Authors:** João M. Parente, Rogério Simoes, Abilio P. Silva, Paulo N. B. Reis

**Affiliations:** 1 C-MAST—Centre for Mechanical and Aerospace Science and Technologies, Universidade da Beira Interior, Rua Marquês d’Avila e Bolama, 6201-001 Covilhã, Portugal; abilio@ubi.pt; 2 FibEnTech, Fiber Materials and Envornmental Technologies, University of Beira Interior, Rua Marquês d’Ávila e Bolama, 6201-001 Covilhã, Portugal; 3 University of Coimbra, CEMMPRE, ARISE, Department of Mechanical Engineering, Rua Luis Reis dos Santos, 3030-788 Coimbra, Portugal

**Keywords:** nanocomposites, epoxy resins, graphene, carbon nanofibers, hybrid nano-reinforcements, curing temperature, manufacturing process, mechanical testing

## Abstract

This study aims to analyze the effect of the curing temperature of nano-reinforcements during the manufacturing process on the mechanical properties of composites involving graphene (GNP), carbon nanofibers (CNFs), and a hybrid mixture of these two nanoparticles. In this context, the type of nanoparticles, their content, their type of resin, and their hybridization were considered. The results showed that both nanoparticles increased the viscosity of the resin suspension, with an increase of between 16.3% and 38.2% for GNP nanoparticles and 45.4% and 74% for CNFs depending on the type of resin. Shrinkage was also affected by the addition of nanoparticles, as the highest results were obtained with GNP nanoparticles, with a 91% increase compared with the neat resin, and the lowest results were obtained with CNFs, with a decrease of 77% compared with the neat resin. A curing temperature of 5 °C promoted the best bending and hardness performance for all composites regardless of the type of resin and reinforcement used, with improvements of up to 24.8% for GNP nanoparticles and 13.52% for CNFs compared with the neat resin at 20 °C. Hybridization led to further improvements in bending properties and hardness compared with single-reinforcement composites due to a synergistic effect. However, the effectiveness of hybridization depends on the type of resin.

## 1. Introduction

Polymeric matrix-based composites (PMCs) are increasingly replacing metallic materials due to their high specific strength and stiffness, good fatigue performance, corrosion resistance, and low processing costs [1,2,3,4]. However, in these materials, the matrix is in the lowest strength phase and any improvement promotes the overall mechanical strength of the composite material. Furthermore, reinforcing the matrix can increase the interfacial strength between the matrix and the fiber (fabric) [5].

Nanoparticles usually have a size between 1 and 100 nm and possess increased chemical activity, unique surface effects, and particular physical properties [6,7]. Several studies have shown that adding low concentrations of nanoparticles leads to increases in mechanical and thermal properties without compromising the density, toughness, or manufacturing process due to the larger surface/interface area per unit volume and the increased physical and chemical interactions between the matrix and nanoparticles [6,8,9]. Among the more common nanoparticles available for use in these types of materials, carbon-based nanoparticles such as carbon nanofibers and graphene nanoplatelets appear to be some of the more promising due to their good mechanical, electrical and thermal properties [10].

Graphene, for example, is a material obtained from the exfoliation of graphite, which naturally exists as a powder that is usually mixed with the matrix of the desired material or deposited on the material’s surface. In its more basic state, graphene is composed of a single layer of carbon atoms, with sp^2^ configuration forming a honeycomb-like structure [11]. It can be subdivided into three groups: graphene nanosheets (GNs), graphene oxide (GO), and graphene nanoplatelets (GNPs). Graphene nanosheets are the basic units of graphene; they consist of single layers of graphene forming a sheet-like surface [12]. Compared with carbon nanotubes, they can offer greater purity and have lower production costs due to the fact that they are synthesized from graphite [13]. Compared with steel, graphene has a higher tensile strength and thermal conductivity but a lower electrical conductivity [12]. The second type, graphene oxide, is composed of graphene nanosheets with added oxy groups. The presence of oxy groups on the graphene’s surface allows for better graphene/matrix interactions due to the more hydrophilic surface that reduces interplanar forces, but this behavior only occurs in aqueous media and is not suitable for most organic polymers [12,14]. These oxy groups can also be used as anchor points for binding molecules of interest in the creation of functionalized graphene [15]. Graphene oxide also possesses tunable electronic properties. For example, it is an electrical insulating material due to the high resistance created by the sp^3^ hybridization of its carbon atoms [16]. However, if graphene oxide is reduced, it can be transformed into a semiconductor or semimetal [17]. The third type, graphene nanoplatelets, consists of several layers of graphene nanosheets connected to each other through van der Waals forces [18]. The size of graphene nanoplatelets varies between 1 and 15 nm in diameter and 5 nm in thickness. This allows them to have larger contact areas between the matrix and nanocomposite, increasing the potential interactions between the matrix and graphene [19]. Similarly, graphene nanosheets can also be functionalized to further increase the interactions between the matrix and graphene [20]. According to the literature, depending on the type of graphene and matrix used, graphene nanosheets can promote significant improvements in mechanical performance, especially at the level of strength and stiffness [21,22,23].

Carbon nanofibers (CNFs) have a similar composition but different overall structures. They usually have a conical (bamboo-like) or cylindrical (herringbone) configuration, stacked between 50 and 200 nm [24,25]. They are characterized by good electrical and thermal conductivity, and, when properly functionalized, they improve the mechanical properties of composites [26,27,28,29].

In addition to the benefits reported in the literature that can be obtained with each of the described nano-reinforcements alone, some studies have suggested using them together. However, most of the published works have addressed this hybridization for use in batteries, and few have focused on its effect on mechanical properties. For example, Shokrieh et al. [30] studied the fatigue response of composites reinforced with graphene and carbon nanofibers. Compared with the results obtained for neat resin, carbon nanofibers (CNFs) and graphene nanoplates (GNPs) improved fatigue life by 24 and 27.4 times, respectively. However, when a hybridization of the two nano-reinforcements (CNF + GNP) in a 1:1 ratio was used, the improvements reached values of around 37.2 times. The authors also observed that while GNPs increased stiffness, CNFs increased strength [30]. Li et al. [31] hybridized carbon nanofibers with graphene oxide (GO) and evaluated their effects on mechanical properties at cryogenic temperatures. They concluded that hybridization gave better results than composites containing only one of the nanoparticles. Papageorgiou et al. [32] hybridized GNPs with short carbon fibers to reinforce a PEEK matrix and, similar to the previous authors, obtained better mechanical properties compared with a single nano-reinforcement. Tian et al. [33] studied the effect of multi-fillers on the response of epoxy resin nanocomposites to hygrothermal aging and found benefits compared with previously reported research due to the hydrophobic and blocking performance of graphite or SCFs (short carbon fibers) and because TiO_2_ (titanium dioxide) or ZnS (zinc sulfide) fillers increase diffusion paths and lead to greater durability.

Regardless of the benefits reported above for the use of nano-reinforcements, the literature also shows that the manufacturing process significantly affects the mechanical properties of composites. For example, Ferreira et al. [34] compared two different types of dispersion processes (direct and indirect) and concluded that the indirect method leads to lower mechanical results due to the presence of residual acetone. In addition, Santos et al. [35] observed that the rotation speed of the mixer, dispersion time, and vacuum time affect the mechanical properties of composites. Uthaman et al. [36] emphasized the enormous tendency of carbon-based nano-reinforcements to form agglomerates due to their weak intermolecular interactions and the consequent weak interfacial strength with polymeric matrices. Therefore, in order to reduce agglomerates, they suggest various mixing/dispersion methods but highlight the use of surfactants to avoid destroying the integrity of the nano-reinforcements.

In this context, it is of the utmost importance to adopt manufacturing procedures whose parameters are the most appropriate for the used resins and nanoparticles. Furthermore, while the literature already provides well-consolidated knowledge for resins filled with single nano-reinforcements (graphene, carbon nanofibers, etc.), the authors do not consider the same knowledge available for the hybridization of nano-reinforcements. Therefore, the main goal of this study was to analyze the effect of the curing temperature during the manufacturing process on the mechanical properties of nanocomposites involving graphene, carbon nanofibers, and a hybrid mixture of these two nano-reinforcements. For this purpose, two epoxy resins were used because composites with epoxy resin matrices based on carbon nano-reinforcements have a strong potential for use in various industries [37,38,39,40]. Both resins have the same chemical base but differ in terms of viscosity, which is why they were selected.

## 2. Materials and Methods

In this study, two epoxy-based matrices were used. Both had bisphenol A (DGEBA) and bisphenol F (DGBF) in their chemical base and different viscosities. The nanocomposites produced with the SR 8100 epoxy resin used the SD 8824 hardener (both components supplied by Sicomin, Châteauneuf-les-Martigues, France) and had a resin viscosity of 285 ± 60 mPa × s at 25 °C, while the nanocomposites produced with the AH 150 epoxy resin used the IP 430 hardener (both supplied by Ebalta, Rothenburg ob der Tauber, Germany) and had a resin viscosity of 250 ± 50 mPa × s at 25 °C. These resins were previously analyzed by the authors, and more details can be found in [41,42,43]. Graphene nanoplatelets supplied by Graphenest (Paradela, Portugal) and carbon nanofibers, supplied by Sigma Aldrich (St. Louis, MO, USA) were used for the nano-reinforcements [44].

The effect of nanoparticles on the resin’s viscosity was evaluated initially. For this purpose, different weight contents of graphene and carbon nanofibers (from 0 wt.% to 1 wt.%), as well as a hybrid mixture involving both nano-reinforcements, were considered. Each resin was mixed at room temperature with the fillers for three hours by ultrasonication and without the presence of the hardener to avoid polymerization and its consequent effects on the measurements. To obtain the viscosity values of the nanocomposites, a Haake RS150 rheometer (Waltham, MA, USA) with a conical plate device (C35/2Ti) was used, and the tests were performed at a controlled temperature of 25 °C and a shear rate of between 0 and 100 s^−1^.

Resin/nanoparticle interactions were evaluated using contact angle tests. For this purpose, 10 mm diameter specimens were produced by compressing graphene and carbon nanofibers. Using the sessile methodology, 5 µL of epoxy resin was dropped on the surface of the specimens, and the contact angle was recorded for 8 s using the DataPhysics OCAH 200 system (Filderstadt, Germany).

**Figure 1:**
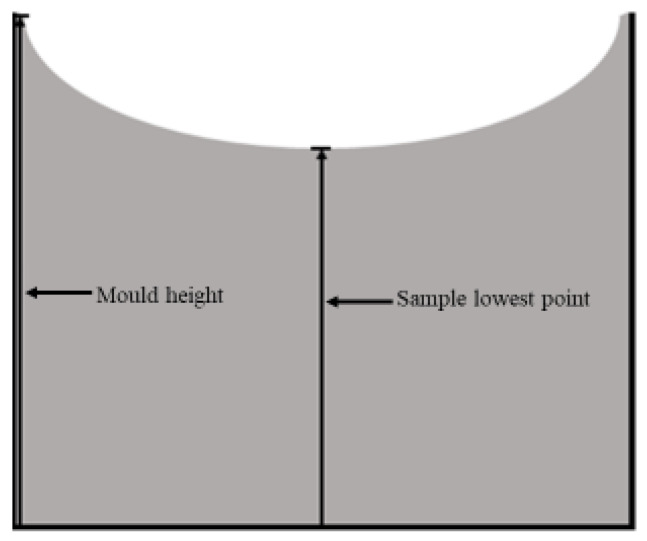
System used to analyze the nanofillers’ effect on the shrinkage behavior.

The shrinkage effect was also analyzed for different configurations: (a) 0, 0.75 and 1 wt.% graphene; (b) 1 wt.% carbon nanofibers; and (c) hybrids with 0.5/0.25, 0.375/0.375 and 0.25/0.5 graphene and carbon nanofibers. For this purpose, the nanoparticles were mixed at room temperature with the resins using a mechanical mixer at 1000 rpm and an ultrasonic bath. This process of mixing and dispersing the nano-reinforcements was carried out for 3 h, and the manufacturing process was completed by adding the hardener to the system using the same mixer at 300 rpm for 5 min. Finally, the system was placed in a vacuum chamber to remove any air bubbles and then poured into molds with a height of 37 mm and diameter of 25 mm. The nano-enhanced resin was poured until it filled the molds (poured to the top). Then, it was cured at room temperature for 24 h and post-cured at 40 °C for 24 h. After cure and post-cure, the height of the central part of the sample was measured and compared with the height of the mold, as shown in Figure 1.

Regarding the effect of curing temperature on mechanical performance, Table 1 summarizes all the analyzed architectures and the weight contents of the different nano-reinforcements (graphene nanoplatelets, carbon nanofibers, and a hybrid reinforcement involving both).

The production process of the specimens was similar to that described above for obtaining the nano-enhanced resins used in the shrinkage study, but different and properly controlled curing temperatures were used. Table 2 summarizes the studied temperatures, and it should be noted that the curing times (24 h for the Sicomin resin and 48 h for the Ebalta resin according to the suppliers) were similar for all of them, and all specimens were subsequently post-cured following the supplier’s recommendations (40 °C for 24 h for the Sicomin resin and 80 °C for 5 h for the Ebalta resin). The dimensions of the specimens used in this study were 120 × 80 × 3 mm^3^. In terms of the hybrid composites, it should be highlighted that all the architectures shown in Table 1 had the same total weight content of nano-reinforcements for each resin, i.e., 0.75 wt.% for the Sicomin resin (0.5 wt.% CNF + 0.25 wt.% GNP, 0.375 wt.% CNF + 0.375 wt.% GNP, and 0.25 wt.% CNF + 0.5 wt.% GNP) and 1 wt.% for the Ebalta resin (0.75 wt.% CNF + 0.25 wt.% GNP, 0.5 wt.% CNF + 0.5 wt.% GNP, and 0.125 wt.% CNF + 0.875 wt.% GNP). These values were obtained from a preliminary study previously carried out to determine the weight contents (wt.%) of nano-reinforcements that maximize the bending properties of each resin. GNP nanoparticles were used for this purpose, and the obtained results are summarized in Table 3. The obtained results are in complete agreement with those reported in studies carried out by Santos et al. [43,45,46], who used the same resins and CNFs as nano-reinforcements. They observed that higher amounts of CNFs promoted agglomerates that significantly affected the mechanical properties. Furthermore, differences in viscosities were not relevant due to the greater physical–chemical compatibility between the Sicomin resin and CNFs; however, the authors also recognized that low-viscosity resins promote better mechanical properties due to the better organization of nanoparticles [45,46]. These arguments also explain the results shown in Table 3.

The mechanical properties evaluated in this study were obtained in the bending mode because, according to the literature [47,48], it is the most sensitive for these purposes. These tests were carried out according to the ISO 178-2004 standard [49] at room temperature using a Shimadzu AG-X universal testing machine (Duisburg, Germany) equipped with a 10 kN load cell. For each condition, 5 specimens with dimensions of 60 × 10 × 3 mm^3^ were used, and all tests were performed at a displacement rate of 2 mm/min and a span of 40 mm. The bending strength was calculated as the nominal stress at the middle span section obtained using the maximum value of the load, while the stiffness modulus was obtained via the linear regression of the load–displacement curves considering the interval in the linear segment with a correlation factor greater than 95%. Subsequently, the fracture surfaces were analyzed with scanning electron microscopy using an SEM VP Hitachi S—3400 N at 20 KV with a resolution of 6.4 nm (Tokyo, Japan). Microhardness was also measured using a Shimadzu HMV-G Vickers microdurometer (Duisburg, Germany). For each condition, 10 measurements were performed with an applied load of 200 g and a holding time of 15 s.

Finally, FTIR tests were also performed to evaluate possible molecular changes in the matrix resulting from the different curing temperatures. For this purpose, a VERTEX 80v FTIR spectrometer (Billerica, MA, USA) was used, with ATR in vacuum and a mid-range IR from 4000 to 400 cm^−1^. For each condition, three samples were used.

## 3. Results

**Figure 2:**
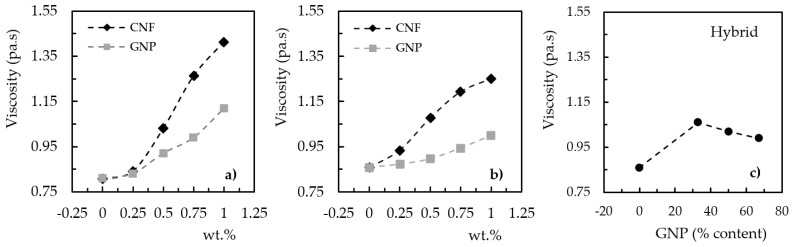
Effect of the nano-reinforcement on the resin suspension viscosity for the (**a**) Ebalta resin, (**b**) Sicomin resin, and (**c**) hybrid mixture.

In terms of viscosity, Figure 2 shows that the values obtained for the viscosities of the neat resins were in line with those provided by the suppliers, but when they were nano-enhanced with graphene nanoplatelets (GNPs) or carbon nanofibers (CNFs), the viscosity of the suspension increased in both cases. It is important to note that, for the hybrid reinforcement, the graphene content on the x-axis represents its percentage in terms of total particles added, i.e., 0% (neat resin), 33% (0.25 wt.% GNP + 0.5 wt.% CNF), 50% (0.375 wt.% GNP + 0.375 wt.% CNF), and 67% (0.5 wt.% GNP + 0.25 wt.% CNF).

For example, compared with the values observed for neat resins, the viscosity increased by around 16.3% (from 0.86 to 1.0 Pa × s) and 38.3% (from 0.81 to 1.12 Pa × s), respectively, for the nano-enhanced Sicomin and Ebalta resins with 1 wt.% of graphene and these values were 45.4% (from 0.86 to 1.25 Pa × s) and 74.1% (from 0.81 to 1.41 Pa × s) for the same carbon nanofiber content. However, despite the increase in viscosity of both resins, the reported values do not affect their use in the production of nanocomposites, as demonstrated by other studies using the same nano-reinforced resins with similar and other nanoparticles [43,45,46,50]. Furthermore, the greater increase in viscosity observed for resins reinforced with carbon nanofibers (CNFs) can be explained by the thin and long structure of the nanoparticles, which allows for greater interactions with the resin due to the higher aspect ratio [45]. On the other hand, graphene nanoplatelets (GNPs) are flat and planar, which, in this case, leads to a high aspect ratio in two dimensions. However, their geometry allows them to slide over each other more easily than CNFs, so the increase in viscosity is not as significant [51,52,53]. On the other hand, the hybrid mixture involving both nano-reinforcements showed that, with the increase in the content of GNPs and consequent decrease in CNFs, the viscosity tended to reach values close to those obtained for the composite reinforced only with graphene. Moreover, these observed results were similar to those found by other authors [30,31,32].

**Figure 3:**
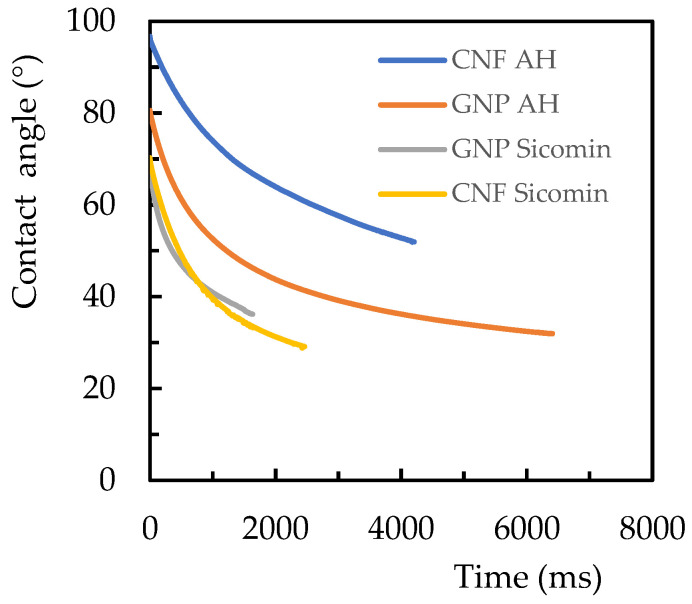
Time profile of the contact angle between the resins and the nanoparticles (CNFs and GNPs).

Regarding the analysis of the contact angles between the resin and nanoparticles, Figure 3 shows their evolution with time for each configuration. It is possible to observe that, regardless of the nano-reinforcement, the contact angles were higher for the Ebalta resin and the Sicomin resin promoted very similar viscosities. For example, the highest contact angles were observed for the Ebalta resin/CNF pair, with initial values of 94° and 52° after 4000 ms, while for the Ebalta resin/GNP pair, these values were 80° and 32°, respectively. The same comparison for the Sicomin resin showed that the contact angles varied from 70° to 34° after 1600 ms with CNFs and from 69° to 37° with GNPs. Therefore, these results reveal that the Sicomin resin has a higher ability to wet both nano-reinforcements, which, according to Santos et al. [43,45], is explained by the difference in polarity between the resins.

Table 4 shows the results obtained from the shrinkage tests, where it is noticeable that the mold height values (H0) were always higher than those observed for the lowest height (H2), which usually occurred in the center of the mold.

It can also be seen that both nano-reinforcements affected the shrinkage of the nanocomposite, though with more significant values for GNPs. For example, the shrinkage value of the neat Sicomin resin was about 3.1%, but when 0.5 wt.% and 1 wt.% of GNPs were added, the percentage variation increased by 76% (from 3.08% to 5.42%) and 91.6% (from 3.08% to 5.9%), respectively. This means that higher shrinkage values were observed. On the other hand, the decrease was only 0.7% when 1 wt.% of CNFs was added to the resin, a value that was 88% lower than that observed for the same weight content of GNPs. In this case, CNFs were responsible for the lower shrinkage values. Finally, when the nano-reinforcements were combined (hybridization), it was possible to observe that lower graphene content (consequently, higher CNF content) promoted lower shrinkage values. For example, the shrinkage of the 0.5 GNP + 0.25 CNF hybrid was 5.7%, which was 85.1% higher than that observed for the neat resin (from 3.08% to 5.7%). However, when the hybridization had values of 0.25 GNP + 0.5 CNF, there was a reduction of around 70.7% (from 5.7% to 1.67%). These results are in line with those of a similar study (using the same methodology) that was performed by Koziol et al. [54], in which 1 wt.% graphene did not lead to a reduction in shrinkage but the same content of carbon nanotubes led to shrinkage. Therefore, the molecular structure of the nanoparticles affects the shrinkage of a nanocomposite, as longer structures lead to a positive effect on the shrinkage of such nano-materials.

**Figure 4:**
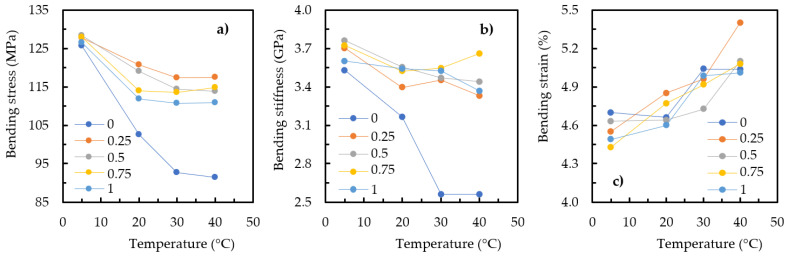
Curing temperature effect for GNP nanocomposites involving the Sicomin resin on (**a**) bending stress, (**b**) bending stiffness, and (**c**) bending strain.

Figure 4 shows the effect of curing temperature during the manufacturing process on the bending properties of the nanocomposites produced with the Sicomin resin and different GNP contents. It should be noted that the aim of this study was to analyze not the direct effect of graphene content on mechanical performance but rather its influence on the effect of curing temperature and, consequently, the mechanical response. Therefore, in this context and considering the curing temperature effect on the neat resin, Figure 4a shows that the highest bending strength was obtained at 5 °C, with an average value of 125.6 MPa; however, when the temperature was increased to 20 °C, it caused a decrease of around 18.4% (from 125.6 to 102.5 MPa). This value increased to 26.2% (from 125.6 to 92.7 MPa) and 27.2% (from 125.6 to 91.4 MPa) when the curing temperature increased to 30 °C and 40 °C, respectively. In terms of nanocomposites, considering the resin reinforced with 0.5 wt.% GNP as an example, a similar effect was observed to that described for the neat resin, though with decreases of around 7.2% (from 128.3 to 119 MPa), 10.8% (from 128.3 to 114.4 MPa), and 11.2% (from 128.3 to 113.9 MPa) for curing temperatures of 20 °C, 30 °C, and 40 °C, respectively. Therefore, it is possible to conclude that, regardless of the GNP content, the highest bending strength always occurred at a curing temperature of 5 °C, and higher values led to decreases in bending strength. Finally, Figure 4b shows an effect very similar to that described previously for bending stiffness, while Figure 4c shows that the bending strain increased with increasing curing temperature, evidencing a more ductile behavior.

**Figure 5:**
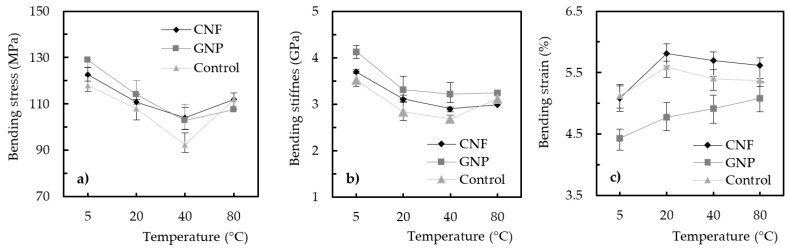
Curing temperature effect on nanocomposites produced with the Ebalta resin and reinforced with 0.75 wt.% GNP and 0.75 wt.% CNF on (**a**) bending stress, (**b**) bending stiffness, and (**c**) bending strain.

A similar study was performed for the nanocomposites produced with the Ebalta resin and both nano-reinforcements (GNPs and CNFs), and Figure 5 shows the curing temperature’s effect on their bending properties. In this case, the studied temperature values were up to 80 °C, and the results are in line with those previously described for the Sicomin resin up to 40 °C. Regardless of the nano-reinforcement used, and similar to what can be observed in Figure 4a, the highest bending strength also occurred for a curing temperature of 5 °C, and its increase up to 40 °C led to lower bending stress values (an average reduction of around 20%). Subsequently, a slight increase was observed with increasing temperatures of up to 80 °C, and the achieved values were very similar to those obtained at 20 °C. Concerning bending stiffness, it was noticed that the highest value was also obtained for the curing temperature of 5 °C, but, in this case, increases in temperature promoted more significant decreases than those observed for bending strength (about 29%). However, this resin has the particularity that, regardless of the increase in temperature, the stiffness of the nanocomposite remained practically constant. Finally, in terms of bending strain, a behavior opposite to that described for stiffness was observed. In this case, it increased from 5 to 20 °C, after which it remained practically constant for higher curing temperature values. Therefore, similar to the Sicomin resin, it was also shown that increasing the curing temperature of the Ebalta resin led to a more ductile behavior of the nanocomposites.

**Figure 6:**
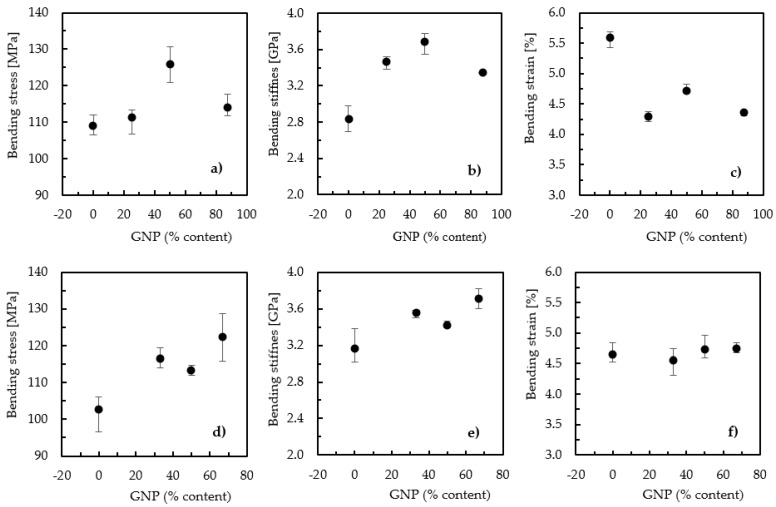
(**a**) Bending stress for nanocomposites with the Ebalta resin; (**b**) bending stiffness for nanocomposites with the Ebalta resin; (**c**) bending strain for nanocomposites with the Ebalta resin; (**d**) bending stress for nanocomposites with the Sicomin resin; (**e**) bending stiffness for nanocomposites with the Sicomin resin; (**f**) bending strain for nanocomposites with the Sicomin resin.

The effect of curing temperature was also studied for the hybrid nanocomposites involving GNPs and CNFs. Before that, however, the content of each nano-reinforcement that maximized the flexural properties of the nanocomposite was determined. It is important to highlight that, in this analysis, a curing temperature of 20 °C was used and the total content of nano-reinforcements was equal to the maximum nanoparticle content that maximized the mechanical properties of each resin (see Table 3), i.e., 0.75 wt.% for the Sicomin resin and 1 wt.% for the Ebalta resin. At this stage, the results will only be compared with each other to determine the ideal combination of each nanoparticle and, subsequently, to determine the real effect of curing temperature on maximizing the properties of the hybrid nanocomposites. Figure 6 shows the results obtained in this study where, as mentioned above, the graphene content on the x-axis represents its percentage of the total particles added, i.e., 0% (neat resin), 25% (0.75 wt.% CNF + 0.25 wt.% GNP), 50% (0.50 wt.% CNF + 0.50 wt.% GNP), and 87.5% (0.125 wt.% CNF + 0.875 wt.% GNP) for the Ebalta resin (Figure 6a–c) and 0% (neat resin), 33% (0.5 wt.% CNF + 0.25 wt.% GNP), 50% (0.375 wt.% CNF + 0.375 wt.% GNP), and 67% (0.25 wt.% CNF + 0.5 wt.% GNP) for the Sicomin resin (Figure 6d–f).

From Figure 6a,b, which shows the hybridization effect for the Ebalta resin, it is possible to observe that both the bending stress and bending modulus reached their highest values for 0.5 wt.% of GNPs and 0.5 wt.% of CNFs (i.e., the same contents of both nano-reinforcements). Compared with the neat resin, the hybridization of the nano-reinforcements led to improvements of around 16.3% (from 109 to 126.8 MPa) in bending stress and 29.9% (from 2.84 to 3.69 GPa) in bending modulus. On the other hand, the bending strain decreased by about 15.6% (from 5.59 to 4.72%) relative to the neat resin, which indicates that the hybrid nanocomposite was less ductile than the neat Ebalta resin. Regarding the Sicomin resin, the highest values of the bending stress and modulus were achieved for 0.5% wt.% of GNPs and 0.25% wt.% of CNFs, which were 19.2% (from 102.8 to 122.5 MPa) and 17% (from 3.17 to 3.71 GPa) higher than those observed for the neat resin. In terms of bending strain, the average value observed for the neat resin was slightly lower than that obtained for the hybrid nanocomposite that maximized the bending strength, but this can be neglected given the observed dispersion. Therefore, unlike the Ebalta resin, the ductility was not affected in nanocomposites produced with the Sicomin resin.

It is possible to conclude from this study that hybridization is beneficial because it promotes (in some cases very considerable) improvements in bending properties compared with those of neat resins. These results are in line with those obtained by Shokrieh et al. [30], who also studied the bending response of nanocomposites with an epoxy matrix reinforced with 0.25 wt.% of graphene and 0.25 wt.% of CNFs. In terms of bending strength, hybridization promoted values of around 123 MPa, while nanocomposites with only 0.25 wt.% graphene and 0.25 wt.% CNF promoted values of 118 MPa and 121 MPa, respectively. However, the benefits found by those authors are lower than those of the present study, probably because the nanoparticle content was not optimized. The bending stiffness followed the same trend, but it was in fatigue strength that the highest benefits were observed. In this case, the hybridization promoted much higher fatigue lives than the neat resin (around 43% higher) because graphene increased the stiffness of the nanocomposite and the pull-out of the CNFs increased the strength. Consequently, multi-nanoparticles led to toughening and the crack propagation in the polymer was significantly slowed down. In addition, the higher surface hardness promoted by the multi-nanoparticles must also be considered.

The results of this study are also similar to those obtained by Yue et al. [55], where an epoxy-based matrix achieved its maximum mechanical performance for 0.8 wt.% CNT (carbon nanotubes) and 0.2 wt.% GNP (graphene). According to the authors, both the bending strength and modulus improved significantly compared with single-filler composites (CNTs and GNPs) due to the synergistic effect of the different nano-reinforcements. For other concentration ratios, it was observed that the data followed the mixing rule. Chatterjee et al. [56]. also found benefits with nanoparticle hybridization in terms of bending response and fracture toughness, which were also explained by the synergistic effects between CNTs and GNPs. Moreover, they also noticed that the GNP particle size had a pronounced effect on the mechanical properties and thermal conductivity of the nanocomposite. Ladani et al. [57] studied the addition of hybrid combinations of CNFs with GNPs to an epoxy resin, and they observed a very significant increase in the quasi-static fracture energy (G_Ic_) due to multiple intrinsic (i.e., interfacial debonding and void growth) and extrinsic (i.e., pull-out and bridging) toughening mechanisms introduced by these nanofillers.

**Figure 7:**
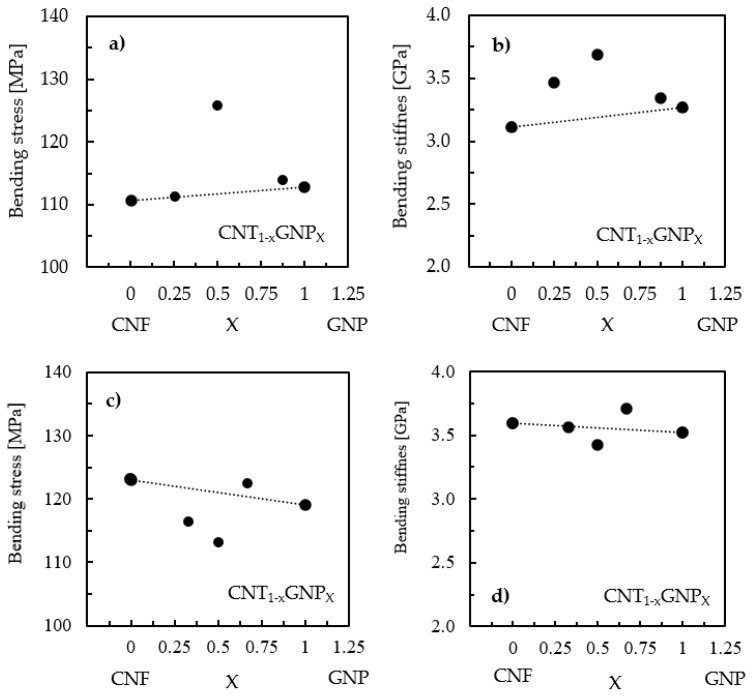
(**a**) Synergistic effect of the bending stress for nanocomposites with the Ebalta resin; (**b**) synergistic effect of the bending stiffness for nanocomposites with the Ebalta resin; (**c**) synergistic effect of the bending stress for nanocomposites with the Sicomin resin; (**d**) synergistic effect of the bending stiffness for nanocomposites with the Sicomin resin.

Based on the previously reported synergistic benefits and using the same schematic representation suggested by Yue et al. [55], Figure 7 shows the synergistic effect observed in this study and its influence on mechanical properties. 

According to the authors, the results above the line mean that the effect is synergistic, the results along the line follow the mixtures’ law, and the results below the line mean that the effect is antagonistic. Therefore, it is possible to observe in Figure 7a that only the nanocomposite produced with the Ebalta resin and reinforced with 50% of both nanoparticles (0.50 wt.% CNF and 0.50 wt.% GNP) had a synergistic effect on bending strength, while the others followed the mixtures’ law. However, at the level of bending stiffness, all of them had a synergistic effect (Figure 7b), although it was also maximized for the same nanoparticle content (50%). Regarding the Sicomin resin, Figure 7c,d shows that only the nanocomposite reinforced with 0.25 wt.% CNF + 0.5 wt.% GNP had a positive effect (synergistic effect) in terms of bending strength and stiffness.

**Figure 8:**
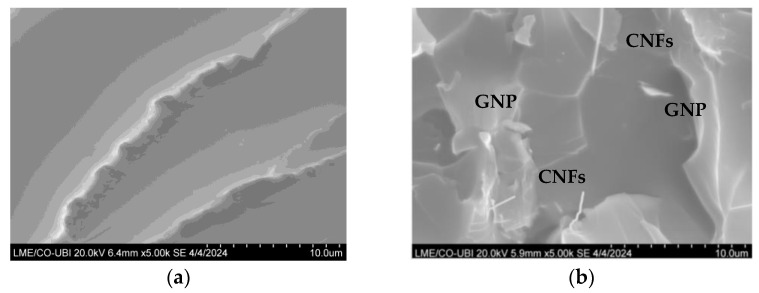
SEM images of the fracture surface relative to the (**a**) neat Ebalta resin and (**b**) hybrid composite involving the Ebalta resin and 50% of both nanoparticles (0.50 wt.% CNF, 0.50 wt.% GNP).

The fracture surfaces of the hybrid and neat nanocomposites were analyzed with scanning electron microscopy (SEM), and Figure 8 shows representative micrographs of each nanocomposite to highlight the benefits of the observed synergistic effect. Figure 8a shows that the neat resin exhibited typical brittle fracture behavior, characterized by long crack propagation with a relatively smooth surface, while Figure 8b reveals the good dispersion of the nanofillers in the matrix, as well as the absence of voids or holes. Therefore, it is clear that both CNFs and GNPs were well dispersed in the resin and that the presence of CNFs prevented the stacking of GNP sheets and the consequent formation of voids.

**Figure 9:**
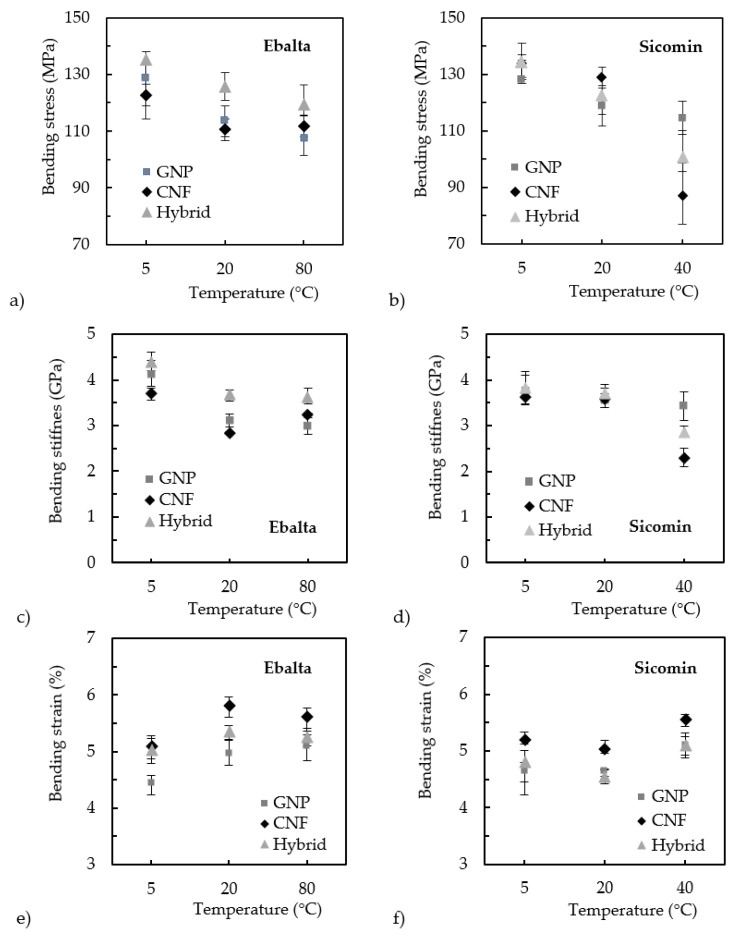
Curing temperature effect for hybrid nanocomposites on (**a**) bending stress and the Ebalta resin, (**b**) bending stress and the Sicomin resin, (**c**) bending strain and the Ebalta resin, (**d**) bending stress and the Sicomin resin, (**e**) bending stiffness and the Sicomin resin, and (**f**) bending strain and the Sicomin resin.

After obtaining the nanofiller content that maximized the bending properties, the curing temperature’s effect on the bending response of these hybrid nanocomposites was analyzed based on the results shown in Figure 9. It should be remembered that this study was carried out on configurations that maximize bending properties, i.e., 0.5 wt.% of GNPs and 0.5 wt.% of CNFs for the Ebalta resin and 0.5% wt.% of GNPs and 0.25% wt.% of CNFs for the Sicomin resin.

Regardless of the resin, the curing temperature had the same effects on the bending properties of the hybrid nanocomposites as those described above for nanocomposites with single reinforcements. For example, in terms of bending stress for all cases analyzed, the maximum observed value was 5 °C and increasing the curing temperature led to its decrease. Compared with those produced at a temperature of 5 °C, the hybrid nanocomposites produced with the Ebalta resin at 80 °C suffered a decrease of around 11.6% (from 135 to 119.4 MPa) in bending strength, while this value was about 25.2% (from 134.3 to 100.5 MPa) for the Sicomin resin at 40 °C. Regarding bending stiffness (see Figure 9c,d), the results showed a similar trend with decreases of the same order of magnitude as those previously observed (17.7% (from 4.4 to 3.62 GPa) and 25.7% (from 3.82 to 2.84 GPa), respectively). Finally, the results shown in Figure 9e,f evidence that, similar to what was observed for the single nano-reinforcements, the hybrid nanocomposites became more ductile as the curing temperature increased. This was a consequence of the increase in the bending strain between the curing temperatures of 5 °C and 80 °C for the hybrid nanocomposites produced with the Ebalta resin by around 5% (from 5 to 5.25%) and by about 6.3% (from 4.8 to 5.1%) for the Sicomin resin between 5 °C and 40 °C. The results in Figure 9 also highlight that, regardless of the curing temperature, the highest bending stress and stiffness values of the Ebalta resin were obtained when it was simultaneously reinforced with GNPs and CNFs, which is in line with results reported in the literature [30,55,56]. However, the nanocomposite reinforced with a single GNP was the one that led to the lowest bending strain results, with the value of the hybrid being very close to that of the CNFs. As mentioned above, the highest increases in bending stress and stiffness can be explained by the synergistic effects resulting from the hybridization of the nanofillers. Similar results were found by Yue et al. [55], whose improvements were explained by the fact that hybridization promotes a more robust nanoparticle network (3D network). In this case, the large surface area of graphene platelets facilitated the dispersion of CNTs, preventing them from re-aggregating, and the CNTs reduced the formation of large-size stacked GNP sheets [55]. In fact, what CNTs do is very efficiently reduce the π–π stacking and agglomeration in GNPs [56]. Therefore, the hybridization of nanofillers with different geometric shapes (high aspect ratios with larger surface areas) is an effective strategy for improving the mechanical properties of nanocomposites because they can contribute to a synergistic effect [56].

However, in addition to this benefit not being as pronounced for the Sicomin resin, it was also more temperature-dependent than in the case of the Ebalta resin. For example, in terms of bending stress at a curing temperature of 5 °C (Figure 9b), hybridization promoted values slightly higher than those observed for single CNF reinforcements (less than 1%) but, subsequently, the increase in temperature led to values intermediate to those of single reinforcements. The same applies to bending stiffness, but, in this case, hybridization promoted values 5.5% and 3.3% higher than those of single CNF reinforcement for curing temperatures of 5 °C and 20 °C, respectively (Figure 9d). It can then be noticed that the stiffness decreases with increasing temperature and, at 40 °C, the value is even intermediate to those obtained with single nano-reinforcements. Finally, the effect of curing temperature on bending strain was very similar to that observed for the Ebalta resin, evidencing a slight increase in ductility with increasing temperature (Figure 9f). These results are in line with those reported in the literature [57,58] when the synergistic effect has not been so evident or has not existed due to the chemical compatibility of the nano-reinforcements and matrices, nano-particle size and shape, etc. [58].

In fact, the Sicomin and Ebalta resins have different characteristics. As observed above (Figure 3), the Sicomin resin has a better ability to wet both nano-reinforcements due to a greater physical–chemical compatibility than the Ebalta resin, i.e., greater acceptance of the nanofillers by the matrix [43,45]. On the other hand, CNFs are relatively easy to disperse into epoxy resins due to their large diameters and are easier to disperse than graphene due to van der Waals interactions between the GNP layers [27,58]. In this context, the greater chemical compatibility between the resin and nano-reinforcements affected their dispersibility and led to the lower synergistic effect reported for the nanocomposites produced with the Sicomin resin.

According to Shokrieh et al. [30], the hybridization of nanoparticles promotes better mechanical properties, including the hardness of the material. Therefore, to complement the analysis carried out in this study, the effect of the curing temperature used during the manufacturing process on the hardness of the hybrid nanocomposites was also studied. The architecture with the nano-reinforcement content that led to the highest bending strength and stiffness in each nanocomposite was used at temperatures of 5, 20, and 80 °C for the Ebalta resin and 5, 20, and 40 °C for the Sicomin resin. Comparing the results only with those of the neat resins, Table 5 shows the average hardness obtained for each condition. These results indicate that the hardness depended on the resin and curing temperature used, confirming what had previously been observed. Furthermore, despite the difference in hardness between neat resins (19.6% for 5 °C), their hybridization always provided higher values regardless of temperature. However, its effect was more effective for the nanocomposites produced with the Ebalta resin, with hardness values of around 2 times higher than those of the Sicomin resin due to the synergistic effect mentioned above. It was also noticed that, as was observed for the bending performance, the highest hardness values were obtained at 5 °C, and subsequent increases in curing temperature led to their decrease. For example, considering the Ebalta resin, the average hardness decreased by around 5.7% and 6.7% when the temperature increased from 5 °C to 20 °C and from 5 °C to 80 °C, respectively, while for the Sicomin resin, these values were about 4.2% (from 5 °C to 20 °C) and 10.8% (from 5 °C to 40 °C), respectively. However, when the hybrid nano-reinforcements (CNFs and GNPs) were added to the resins, these values were about 10% and 13.5% for the Ebalta resin and 4.9% and 12.6% for the Sicomin resin, respectively. These results are in line with those obtained in the bending tests, where the percentages of decrease were, inclusively, of the same order of magnitude.

According to Uzay et al. [59], these higher hardness values are due to the unique properties and combined effects of GNPs and CNFs. In fact, this hybridization promotes a stronger and more evenly distributed reinforcement phase, in which the nano-reinforcements increase the stiffness of the cured epoxy network, thus leading to greater resistance to the deformation of the matrix [54,60]. Therefore, in this context, molecular chain mobility is more restricted, and the nanocomposite is more resistant to the deformation caused by a load [61,62]. However, although most studies have reported increases in hardness with the addition of nano-reinforcements, some of them have reached opposite conclusions. This is especially true for high concentrations of nanoparticles due to the formation of agglomerates (considered defects) and consequent differences in the intensity of the polymerization reaction compared with neat resins [54,63].

**Figure 10:**
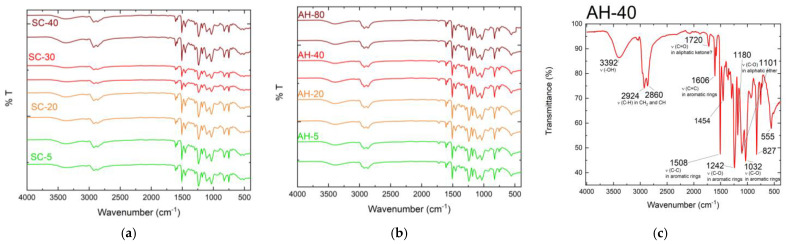
FTIR results for (**a**) the Sicomin resin and (**b**) the Ebalta AH resin. (**c**) Description of each peak for the FTIR results.

Therefore, it can be concluded that curing temperature has a significant effect on the mechanical properties of nanocomposites and, to analyze possible changes that may be caused in the molecular structure of the matrices, an FTIR analysis was carried out. The results are shown in Figure 10, and it should be noted that they only report what was observed for the neat resins.

Based in Figure 10a,b, it is obvious that both resins do not present evident changes in terms of the number of peaks or their intensity, which leads to the conclusion that there were no changes in the molecular structure of the matrices caused by the different curing temperatures. Considering Figure 4c for the Ebalta resin cured at 40 °C as an example, it can be seen that both resins showed peaks at 2924 and 2860 cm^−1^ corresponding to C-H bonds, at 1720 cm^−1^ related to aliphatic acetone, between 1606 and 1032 cm^−1^ various peaks related to C-C and C-O bonds, and, finally, at 1101 cm^−1^ related to aliphatic ether. These results are in line with those in the literature [64,65,66], which has shown the absorption band located at 905 cm^−1^ determines the resin curing/cross-linking degree in terms of epoxy groups [33]. Therefore, the mechanical performance of a nanocomposite depends on the resin curing/cross-linking degree, where higher values correspond to better interfacial strength between the nanofillers and resin. However, the reaction mechanisms that occur in the resin-curing process are influenced by the reaction temperature and curing time, and the ideal values for these parameters are established when an adequate degree of conversion is achieved in terms of epoxy groups [33]. According to Seretis et al. [36], very high values of temperature or time affect the mechanical performance of nanocomposites due to the different thermal expansion coefficients of their constituents and consequent degradation of their interfaces. Moreover, Prolongo et al. [67] found that higher contents of GNPs (5 wt.%) accelerate the curing reaction, but this catalytic effect is not detected for low contents (1 mass%) due to the higher thermal conductivity of the nanocomposite. It was also observed that the curing reaction becomes less exothermic with the introduction of GNPs and the T_g_ of the nanocomposites decreases because hindering the epoxy–amine reaction leads to less perfect networks than those of neat resins. In the study carried out by Rehman et al. [68], it was also noticed that the degree of curing increased with GNP content.

Therefore, because the FTIR analysis did not evidence any changes in the molecular structure of the matrices, the effect observed on the mechanical properties can be explained by the fact that low temperatures lead to a slower and more uniform polymerization/crosslinking process, leading to a more robust 3D network.

## 4. Conclusions

The main goal of this study was to analyze the effect of the curing temperature of nano-reinforcements on the mechanical properties of composites involving graphene, carbon nanofibers, and a hybrid mixture of these two nano-reinforcements. For this purpose, two epoxy resins with different viscosities were used, and some preliminary parameters, such as the viscosity of the nano-reinforced resin suspensions and their shrinkage, were analyzed to better understand the effect of the resin on the final manufacturing process and mechanical performance.

It was observed that both the GNP and CNF nanoparticles increased the viscosity of the resins, though the CNF nanoparticles had more significant effects, with increases of between 16.3% and 38.2% for GNPs and 45.4% and 74% for CNFs combined with the Sicomin and Ebalta resins, respectively. The contact angle between the resins and nanoparticles was higher for the Ebalta resin, indicating better wetting compared with the Sicomin resin. Nanoparticle addition affected shrinkage, with GNPs leading to a 91% increase and CNFs leading to a 77% decrease compared with neat resin. The optimization of hybridization ratios can be used to optimize shrinkage behavior.

Curing temperature had a significant effect on the bending properties of both single and hybrid nanocomposites. Regardless of the resin or reinforcement, the highest bending strength, stiffness, and strain were achieved at the lowest curing temperature (5 °C), with an improvement of up to 24.8% for graphene and 13.52% for carbon nanofibers compared with neat resin at 20 °C, and decreases were observed with increasing temperature. In addition, hybridization can improve the flexural properties of nanocomposites if the synergistic effect of the different nano-reinforcements occurs. However, the resin type proved to be a determining factor in hybridization effectiveness. The hardness tests led to results similar to those observed for the bending response. FTIR analysis showed that there were no significant changes in the molecular structures of the resins at the different curing temperatures.

## Figures and Tables

**Table 1:** Different nanocomposites analyzed in this study.

Resin	Nanoparticles	Weight Content (wt.%)								
		Hybrid Nanocomposites			Single Nanocomposites					Neat Resin
Sicomin	CNFs	0.5	0.375	0.25	0	0	0	0	0.75	0
	GNPs	0.25	0.375	0.5	0.25	0.5	0.75	1	0	0
Ebalta	CNFs	0.75	0.5	0.125	0	0.75	-	-	-	0
	GNPs	0.25	0.5	0.875	0.75	0	-	-	-	0

**Table 2:** Curing and post-curing parameters that were analyzed.

Resin	Curing Process		Post-Curing Process	
	Time (h)	Temperature (°C)	Time (h)	Temperature (°C)
Sicomin	24	5	24	40
		20		
		30		
		40		
Ebalta	48	5	5	80
		20		
		40		
		80		

**Table 3:** Effect of GNP content on bending properties.

Resin	wt.% GNP	Bending Stress [MPa]	Bending Stiffness [GPa]	Bending Strain [%]
Ebalta	0	102.4 (2.41)	2.88 (0.15)	5.05 (0.11)
	0.5	104.3 (4.74)	2.93 (0.17)	5.00 (0.06)
	0.75	107.2 (6.28)	3.01 (0.35)	4.76 (0.61)
	1	111.5 (1.71)	3.27 (0.08)	4.38 (0.21)
	1.5	102.3 (4.54)	2.99 (0.23)	3.24 (0.35)
Sicomin	0	108.9 (6.48)	3.17 (0.16)	4.66 (0.12)
	0.25	114.0 (6.7)	3.39 (0.11)	4.85 (0.05)
	0.5	119.1 (5.69)	3.55 (0.16)	4.41 (0.53)
	0.75	120.7 (6.09)	3.52 (0.28)	4.37 (0.29)
	1	111.9 (5.5)	3.54 (0.05)	3.31 (0.09)

() standard deviation.

**Table 4:** Shrinkage values for hybrid nanocomposites produced with the Sicomin resin.

% Nanoparticles		Mold Height H0 (mm)	Lowest Point H2 (mm)	PV (%)
GNP	CNF			
0	0	37.96	36.79	3.08
0.5	0	37.29	35.27	5.42
1	0	37.15	35.08	5.90
0.5	0.25	37.81	35.75	5.76
0.375	0.375	37.45	36.54	2.49
0.25	0.5	36.63	36.03	1.67
0	1	37.31	37.05	0.70

PV = Percentage Variation = ((H0 − H2) × 100)/H0.

**Table 5:** Effect of the curing temperature on the average hardness.

Resin	Nanoparticles	Average Microhardness (HV)			
		5 °C	20 °C	40 °C	80 °C
Sicomin	-	16.6 (0.35)	15.9 (0.21)	14.8 (0.51)	-
	0.5 GNP + 0.25 CNF	18.2 (0.48)	17.3 (0.19)	15.9 (0.33)	-
Ebalta	-	19.4 (0.69)	18.3 (0.49)	-	18.1 (0.52)
	0.5 GNP + 0.5 CNF	22.9 (0.74)	20.6 (0.30)	-	19.8 (0.25)

(0.XX) = Std Dev.

## Data Availability

Data are contained within the article.
